# Depression-like Behavior Induced by Nesfatin-1 in Rats: Involvement of Increased Immune Activation and Imbalance of Synaptic Vesicle Proteins

**DOI:** 10.3389/fnins.2015.00429

**Published:** 2015-11-10

**Authors:** Jin-Fang Ge, Ya-Yun Xu, Gan Qin, Yao-Nan Peng, Chao-Feng Zhang, Xing-Rui Liu, Li-Chuan Liang, Zhong-Zheng Wang, Fei-Hu Chen

**Affiliations:** ^1^Anhui Key Laboratory of Bioactivity of Natural Products, School of Pharmacy, Anhui Medical UniversityAnhui, China; ^2^Department of Clinical Medicine, The Second Clinical College of Anhui Medical UniversityAnhui, China

**Keywords:** depression, nesfatin-1, hypothalamic-pituitary-adrenal axis (HPA), synapsin I, synaptotagmin I, C-reactive protein (CRP), interleukin 6 (IL-6)

## Abstract

Depression is a multicausal disorder and has been associated with metabolism regulation and immuno-inflammatory reaction. The anorectic molecule nesfatin-1 has recently been characterized as a potential mood regulator, but its precise effect on depression and the possible mechanisms remain unknown, especially when given peripherally. In the present study, nesfatin-1 was intraperitoneally injected to the rats and the depression-like behavior and activity of the hypothalamic-pituitary-adrenal (HPA) axis were evaluated. The plasma concentrations of nesfatin-1, interleukin 6 (IL-6), and C-reactive protein (CRP); and the hypothalamic expression levels of nesfatin-1, synapsin I, and synaptotagmin I mRNA were evaluated in nesfatin-1 chronically treated rats. The results showed that both acute and chronic administration of nesfatin-1 increased immobility in the forced swimming test (FST), and resulted in the hyperactivity of HPA axis, as indicated by the increase of plasma corticosterone concentration and hypothalamic expression of corticotropin-releasing hormone (CRH) mRNA. Moreover, after chronic nesfatin-1 administration, the rats exhibited decreased activity and exploratory behavior in the open field test (OFT) and increased mRNA expression of synapsin I and synaptotagmin I in the hypothalamus. Furthermore, chronic administration of nesfatin-1 elevated plasma concentrations of IL-6 and CRP, which were positively correlated with despair behavior, plasma corticosterone level, and the hypothalamic mRNA expression of synapsin I and synaptotagmin I. These results indicated that exogenous nesfatin-1 could induce the immune-inflammatory activation, which might be a central hug linking the depression-like behavior and the imbalanced mRNA expression of synaptic vesicle proteins in the hypothalamus.

## Introduction

As a metabolic disorder, depression has been associated with anorexigenic or orexigenic peptides (Lang and Borgwardt, 2013; Bali et al., 2014). Nesfatin-1 is a newly discovered anorexigenic factors that cleaved from its precursor nucleobindin 2 (NUCB2; Oh et al., 2006; Stengel et al., 2009). Based on the findings that nesfatin-1 is distributed in the stress-related brain regions, including the hypothalamus (Oh et al., 2006; Brailoiu et al., 2007; Price et al., 2008) and co-localized with stress-related substances (Oh et al., 2006; Kohno et al., 2008; Goebel-Stengel and Wang, 2013), attention has been focused on finding a possible link between nesfatin-1 and depression (Yoshida et al., 2010; Emmerzaal and Kozicz, 2013). It has been reported that the plasma nesfatin-1 level is statistically higher in patients with major depressive disorder (Ari et al., 2011) and associated with the elevated scores of anxiety and depression (Ari et al., 2011; Hofmann et al., 2013), and intracerebroventricular (ICV) injection of nesfatin-1 could induce anxiety- and fear-related behaviors (Merali et al., 2008). Results of our previous study showed that acute stress could increase the plasma concentration and hypothalamic mRNA expression of NUCB2/nesfatin-1 in rats (Xu et al., 2015). However, the causality between nesfatin-1 and anxiety or depression-like changes remains unknown, and little is known about the effect of nesfatin-1 on the neuropsychic behaviors when given peripherally.

Although a specific nesfatin-1 receptor has yet to be identified and thus the actual site of action of this peptide has not yet been determined, compelling evidences, especially the fact that co-localization of nesfatin-1 with corticotropin-releasing hormone (CRH) in the paraventricular nucleus (PVN; Foo et al., 2008), suggest that the physiological effects of nesfatin-1 are associated with the hypothalamic-pituitary-adrenal (HPA) axis. It has also been reported that ICV administration of nesfatin-1 could elevate the plasma levels of adrenocorticotrop hormone (ACTH) and corticosterone (CORT) in rats (Konczol et al., 2010; Yoshida et al., 2010), the mechanism of which might be mediated by its effect on hypothalamic CRH neurons (Yoshida et al., 2010). However, there is no report about the effect of peripherally administered nesfatin-1 on the HPA axis, which has long been taken as one important factor that triggers the depressive episodes.

Synaptotagmin and synapsin are major component proteins of the synaptic vesicle membrane and are required for vesicle fusion and neurotransmitter release (Thome et al., 2001). Our previous study demonstrated that the mRNA expressions levels of synaptotagmin I and synapsin I were both increased in the hypothalami of chronically stressed rats (Ge et al., 2013). Consistent with these findings, stress-induced changes in these two proteins in the hippocampus or cortex after chronic stress have also been observed in other studies (Thome et al., 2001; Wu et al., 2007). Moreover, it has also been reported that antidepressant treatment can alleviate depressive symptoms by inhibiting their expression (Wu et al., 2007; Dagyte et al., 2011; Müller et al., 2011). Given the effect of synaptic vesicle proteins on the pathogenesis of depression and their interaction with anorexigenic or orexigenic neurotransmitters (Fried et al., 1995; Papke et al., 2012; Poretti et al., 2015), one of the aims of the present study is to investigate the potential effect of nesfatin-1 on the expression of synaptotagmin I and synapsin I in the hypothalamus.

Depression as an inflammatory disorder is mediated by pro-inflammatory cytokines (Lang and Borgwardt, 2013; Maes et al., 2014). Meta-analyses have indicated that the most robust evidence-based inflammatory markers associated with depression include interleukin-6 (IL-6), C-reactive protein (CRP), and TNF-a (Dowlati et al., 2010). An increasing number of studies have demonstrated that elevated levels of plasma IL-6 and CRP levels are associated with an increased risk of depression (Liu et al., 2014; Wium-Andersen et al., 2014), and can even predict subsequent depressive symptoms (Valkanova et al., 2013), although no significant correlation between IL-6 or CRP and depression has also been reported (Chocano-Bedoya et al., 2014). Considering the changes of the production of pro-inflammatory cytokines and neuropeptides associated with energy and metabolism regulation in depression (Martinez et al., 2012; Wang et al., 2015), another objective of this study is to investigate whether the immune-inflammatory response is a factor involved in the association between nesfatin-1 and depression.

To gain further insights into the association between nesfatin-1 and depression, nesfatin-1 was administered to rats with a single dose or chronically in the present study, and the depression-like behavior and activity of the HPA axis were evaluated. Additionally, the plasma nesfatin-1concentration and the hypothalamic expression of NUCB2/nesfatin-1 mRNA were examined in nesfatin-1 chronically treated rats. Moreover, the plasma concentrations of CRP and IL-6 and the mRNA expression of synapsin I and synaptotagmin I in the hypothalamus were also evaluated in nesfatin-1 chronically treated rats.

## Experimental procedures

### Animals and drugs

Male Sprague-Dawley (SD) rats, 2 months of age, were purchased from Anhui Experimental Animal Center of China. The rats were maintained under a 12:12 h light: dark cycle (lights on at 07:00 h), housed 3-4 per cage (43 cm length × 31 cm width × 19 cm height) with access to food and water *ad libitum*. The ambient temperature was maintained at 21–22°C with 50–60% relative humidity. All the experimental procedures in this study were approved by the Animal Care and Use Committee at Anhui Medical University, which complies with the National Institute of Health Guide for the Care and Use of Laboratory Animals (NIH publication No. 85-23, revised 1985).

Nesfatin-1 purchased from Phoenix Pharmaceuticals, Inc. (Burlingame, CA, USA) was dissolved in sterile saline solution (0.9% w/v sodium chloride) and administered to the rats by intraperitoneal (i.p.) injection.

### Experiment 1: Single-dose administration of nesfatin-1

Twenty-four SD rats were randomly divided into four groups including control and nesfatin-1(10, 20, 40 μg/kg) groups. Approximately 30 min after the i.p. injection of sterile saline solution or nesfatin-1 at the corresponding dose, a 5-min FST was conducted by placing each rat in a cylinder (height: 60 cm; diameter: 25 cm) containing water at 22 ± 2°C and a depth of 30 cm. The rats were anesthetized and decapitated immediately after the FST.

### Experiment 2: Chronic administration of nesfatin-1

**Figure 1:**
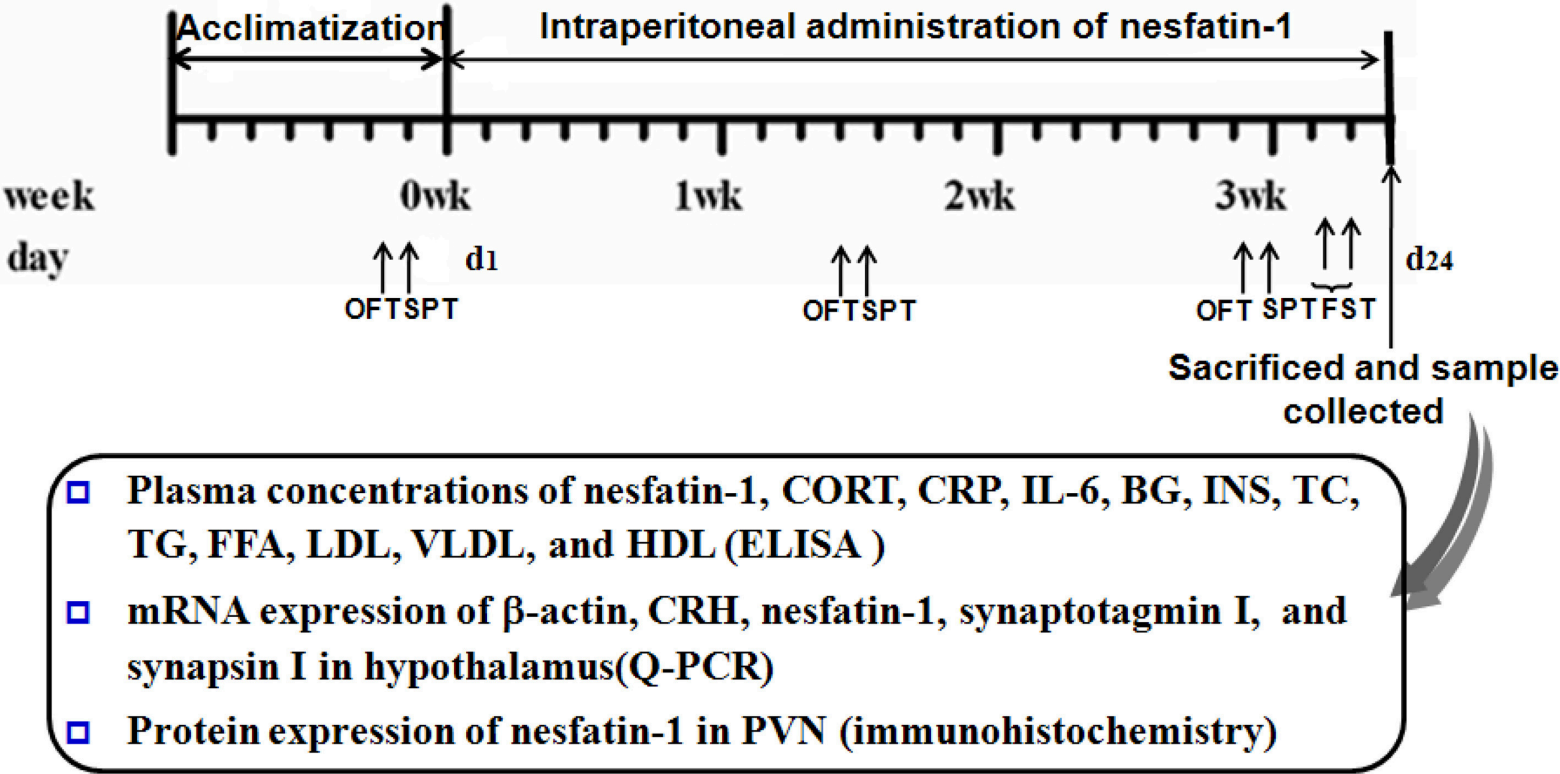
**Scheme of experiment 2**. Annotations: OFT, open-field test; SPT, sucrose preference test; FST, forced swimming test; CORT, corticosterone; IL-6, interleukin-6; CRP, C-reactive protein; BG, Blood glucose; INS, insulin; TC, total cholesterol; TG, triglycerides; FFA, free fatty acid; LDL, low density lipoprotein; VLDL, very low density lipoprotein; HDL, high density lipoprotein.

Forty SD rats were divided into 4 groups matching based on the bodyweight and the result of the first open field test (OFT). The groups included a control and nesfatin-1(10, 20, 40 μg/kg) groups. All the rats received a daily injection of sterile saline solution or nesfatin-1 at the corresponding dose. The animals were continuously treated between 0800 and 1000 h for 3 weeks before the behavioral test. Figure 1 shows the scheme of chronic administration of nesfatin-1.

### Behavioral tests

Behavioral tests were performed in a soundproof room with a neutral environment. All of the tests were conducted between 0830 and 1430, with matching between the groups. The observers were blind to the treatment. The behavioral tests were monitored and recorded by a digital camera interfaced with a computer running the ANY-maze video imaging software (Stoelting Co, Wood Dale, USA).

#### Open-field test

The open-field test was conducted before and on day 10 and 20 of the treatment period. The apparatus consisted of a black square arena 100 × 100 cm, with a black wall 30 cm high. The floor was marked with a grid that divided the floor into 16 equal-sized squares. During a 5-min observation period, the rats in experiment 2 were placed at one corner of the apparatus facing the wall. Total moving distance, duration in the center, and the frequencies of rearing and grooming were recorded.

#### Sucrose preference test

A sucrose preference test was also carried out before, during, and after nesfatin-1 administration. After a 12-h period of food and water deprivation, all the animals in experiment 2 were given free access to two bottles containing plain water and a 2% sucrose solution, respectively. 6 h later, the volumes of water and sucrose consumed were measured. The percentage of sucrose solution compared with the total liquid ingested was used as a measure of the rats' sensitivity to reward.

#### Forced swimming test citation(FST)

The FST was carried conducted according to the method described in our previous study (Ge et al., 2013). The behavioral cylinder was 60 cm high, 25 cm in diameter, filled with 30 cm of water and maintained at 22 ± 2 °C. The FST paradigm included 2 phases: an initial 15 min pre-test followed by a 5 min test 24 h later. Rats were considered to be immobile when they did not make any active movements. Struggling was considered to be the rats making active movements with their forepaws in and out of the water along the side of the swim chamber. Swimming was considered to be the rats making active swimming or circular movements.

### Measurement of the plasma concentrations of CORT, nesfatin-1, IL-6, and CRP

Immediately after the FST in experiment 1 or 24 h after the last behavioral test in experiment 2, the rats were deeply anesthetized with chloral hydrate and the blood was taken from the abdominal aorta. The plasma was collected and the concentrations of CORT, nesfatin-1, IL-6, and CRP were measured using commercially available Enzyme-Linked Immunosorbent Assay (ELISA) kits (CORT: Enzo Life Sciences, Inc., USA; nesfatin-1: Cusabio Biotech. Co., LTD, Wuhan, Hubei, China; CRP and IL-6: Yuanye Biotech. Co., LTD, Shanghai, China) according to the manufacturer's instruction.

### RNA isolation and real time PCR

After blood collection, five rats in each group were selected randomly to collect the hypothalamus and hippocampus to test the parameters selected for the present study, and the samples of the other rats in experiment 2 were used to evaluate the other parameters. The hypothalami were rapidly dissected and frozen quickly in liquid nitrogen, and stored at -80°C. Total RNA was extracted using the TRIzol (Invitrogen, Carlsbad, CA) method. cDNA was synthesized using reverse transcriptase (Promega, Wisconsin, USA). Q-PCR was performed using the SYBR Green PCR Kit (Applied Biosystems, USA) and an ABI Prism 7000 Sequence Detector system in a 25 μL volume for 40 cycles (15 s at 95°C; 60 s at 62°C for rat β-actin, CRH, NUCB2/nesfatin-1, synaptotagmin I, and synapsin I). The primers used in our study were as follows: rat β-actin 5′-TTGCTGACAGGA TGCAGAA-3′ and 5′-ACCAATCCACAC AGAGTACTT-3′; rat CRH 5′-CAGAACAACAGT GCGGGCTCA-3′ and 5′-AAGGCAGACAGGGCG ACAGAG-3′; rat NUCB2 /nesfatin-1 5′- CAAGGG AAAAATCTGGGCCG -3′ and 5′- ACAGTACCG TGCTTGGATGG -3′; rat synaptotagmin I 5′-CTTGAG CAACCAACATCCGC-3′ and 5′-ATGACTGGCACT CACCATTTTT-3′; rat synapsin I 5′-GTTCTTCGG AATGGGGTCAAA-3′ and 5′-GAACCATCT GGGCAAACACC-3′. The relative amount of the target gene was calculated using the 2^−ΔΔCt^ method, and the result from each sample was normalized against that of the β-actin.

### Statistical analysis

All statistical analyses were performed using SPSS (Statistical Package for the Social Sciences) version 12.0.1 (SPSS Inc., Chicago, IL, USA). Data are expressed as the mean ± S.E.M., and *P* < 0.05 was considered statistically significant. Between-group effects on body weight and the performance in the OFT and sucrose preference were analyzed using a Two-way repeated-measure ANOVA with group as between factor and day as within factor followed by a least significant difference (LSD). Statistical analyses of between-group effects on behavioral performance, plasma concentrations of CORT, nesfatin-1, IL-6, and CBP, and the hypothalamic mRNA expression of CRH, NUCB2/nesfatin-1, synaptotagmin I, and synapsin I were conducted using ANOVA followed by LSD *post hoc* tests. A correlation analysis was performed using the Pearson correlation test or the Spearman correlation test when necessary.

## Results

### Increased immobility in the FST and hyperactivity of the HPA axis induced by a single dose of nesfatin-1

**Figure 2:**
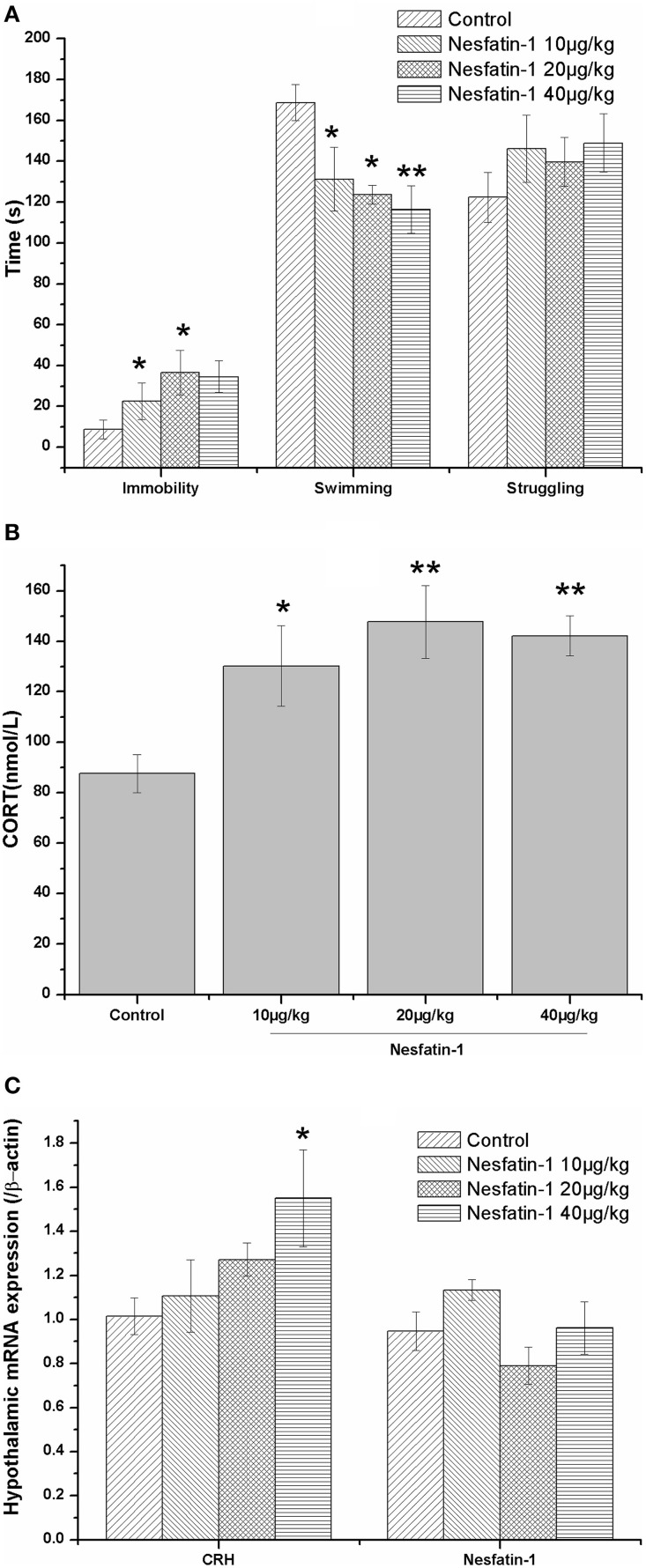
**Effect of a single dose of nesfatin-1 on the immobility in the FST, the HPA axis, and the hypothalamic mRNA expression of nesfatin-1 in rats**. The data are presented as the mean ± SEM, with *n* = 6 for each group. The immobile time was increased and the swimming time was decreased in the nesfatin-1-treated rats compared with the controls **(A)**. The nesfatin-1-treated rats exhibited hyperactivity of the HPA axis, as indicated by the elevated serum CORT concentration **(B)** and CRH mRNA expression in the hypothalamus **(B)**. The mRNA expression of nesfatin-1 in the hypothalamus was not significantly different between the nesfatin-1-treated rats and the controls **(C)**. ^*^P < 0.05 and ^**^P < 0.001 compared with the control group.

As shown in Figure 2A, a single dose of nesfatin-1 (20, 40 μg/kg) significantly increased the immobility of rats in the FST (*F* = 2.369, *P* = 0.109); nesfatin-1 (10, 20, 40 μg/kg) also decreased the swimming time (*F* = 4.606, *P* = 0.017). Consistent with this result, the plasma CORT concentration was increased in the rats administered a single dose of nesfatin-1 (Figure 2B; *F* = 5.456, *P* = 0.009), and the CRH mRNA expression was significantly increased in the hypothalami of the rats in the nesfatin-1 (40 μg/kg) group (Figure 2C) compared with the controls. As shown in Figure 2C, there was no significant difference in the mRNA expression of NUCB2/nesfatin-1 in the hypothalami among the four groups (*F* = 2.504, *P* = 0.096).

### Depression-like behavior induced by the chronic administration of nesfatin-1

The analysis of the bodyweight using a repeated measures ANOVA showed that the training days (*F* = 102.706, *P* < 0.001), but not exposure to nesfatin-1 (*F* = 0.048, *P* = 0.986) affected the bodyweight, with an interactive effect (*F* = 4919.133, *P* < 0.001).

**Figure 3:**
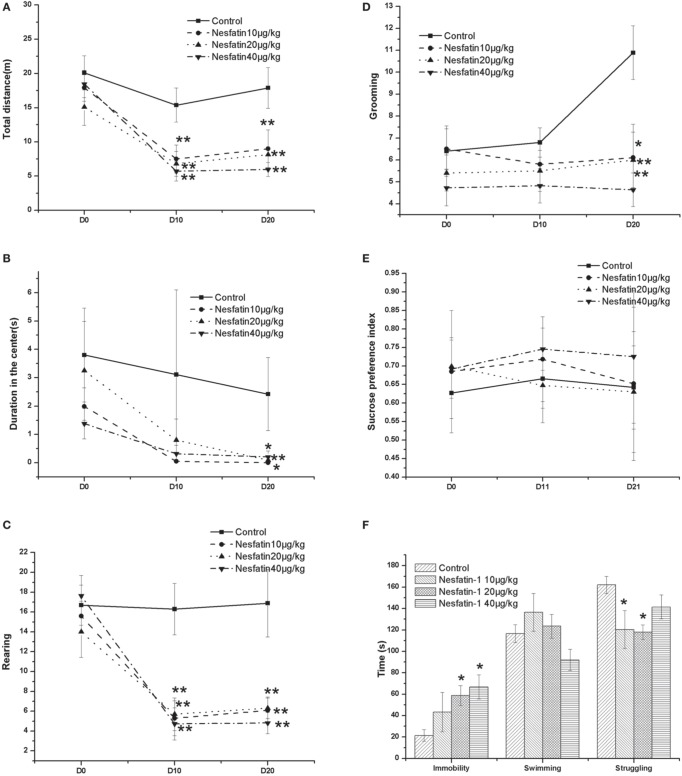
**Effect of chronic nesfatin-1 administration on the performance of rats in the open field test (OFT), FST and sucrose preference test**. The data are presented as the mean ± SEM, with *n* = 10 for each group. In the OFT, both the time and nesfatin-1 treatment affected the total moving distance **(A)**, the number of rearing **(C)**, and the number of grooming **(D)**. A time effect was also found for the duration in the center **(B)**. In the sucrose preference test, neither the time nor the nesfatin-1 treatment affected the sucrose preference index **(E)**. In the FST, after 3-we-eks period of nesfatin-1 administration, the rats spent a longer time immobile and less time struggling **(F)**. ^*^P < 0.05 and ^**^P < 0.001 compared with the control group.

Figure 3 shows the changes in the behavior test results induced by the chronic administration of nesfatin-1. In the OFT (Figures 3A–D), the results showed that both the time and nesfatin-1 treatment affected the total moving distance (time effect: *F* = 17.513, *P* < 0.001; treatment effect: *F* = 6.060, *P* = 0.002; interaction effect: *F* = 283.130, *P* < 0.001), the frequency of rearing (time effect: *F* = 20.054, *P* < 0.001; treatment effect: *F* = 7.554, *P* < 0.001; interaction effect: *F* = 246.839, *P* < 0.001), and the frequency of grooming (time effect: *F* = 2.445, *P* < 0.001; treatment effect: *F* = 4.031, *P* = 0.014; interaction effect: *F* = 326.496, *P* < 0.001). A time effect was also found for the duration in the center (time effect: *F* = 4.707, *P* = 0.015; treatment effect: *F* = 1.887, *P* = 0.149; interaction effect: *F* = 11.405, *P* = 0.002).

In the sucrose preference test (Figure 3E), our results showed that neither the time nor the nesfatin-1 treatment affected the sucrose preference index (time effect: *F* = 0.393, *P* = 0.678; treatment effect: *F* = 1.852, *P* = 0.155; interaction effect: *F* = 2958.698, *P* < 0.001).

In the FST, after the 3-weeks period of nesfatin-1 administration, the rats spent a longer time immobile and less time struggling (Figure 3F).

Interestingly, the results of the Spearman correlation analysis showed that the dose of nesfatin-1 administered was positively correlated with the immobility in the FST (*r* = 0.589, *P* < 0.001) but negatively correlated with the frequencies of rearing (*r* = −0.502, *P* = 0.001) and grooming (*r* = −0.450, *P* = 0.004) in the last OFT.

### Hyperactivity of the HPA axis induced by the chronic administration of nesfatin-1

**Figure 4:**
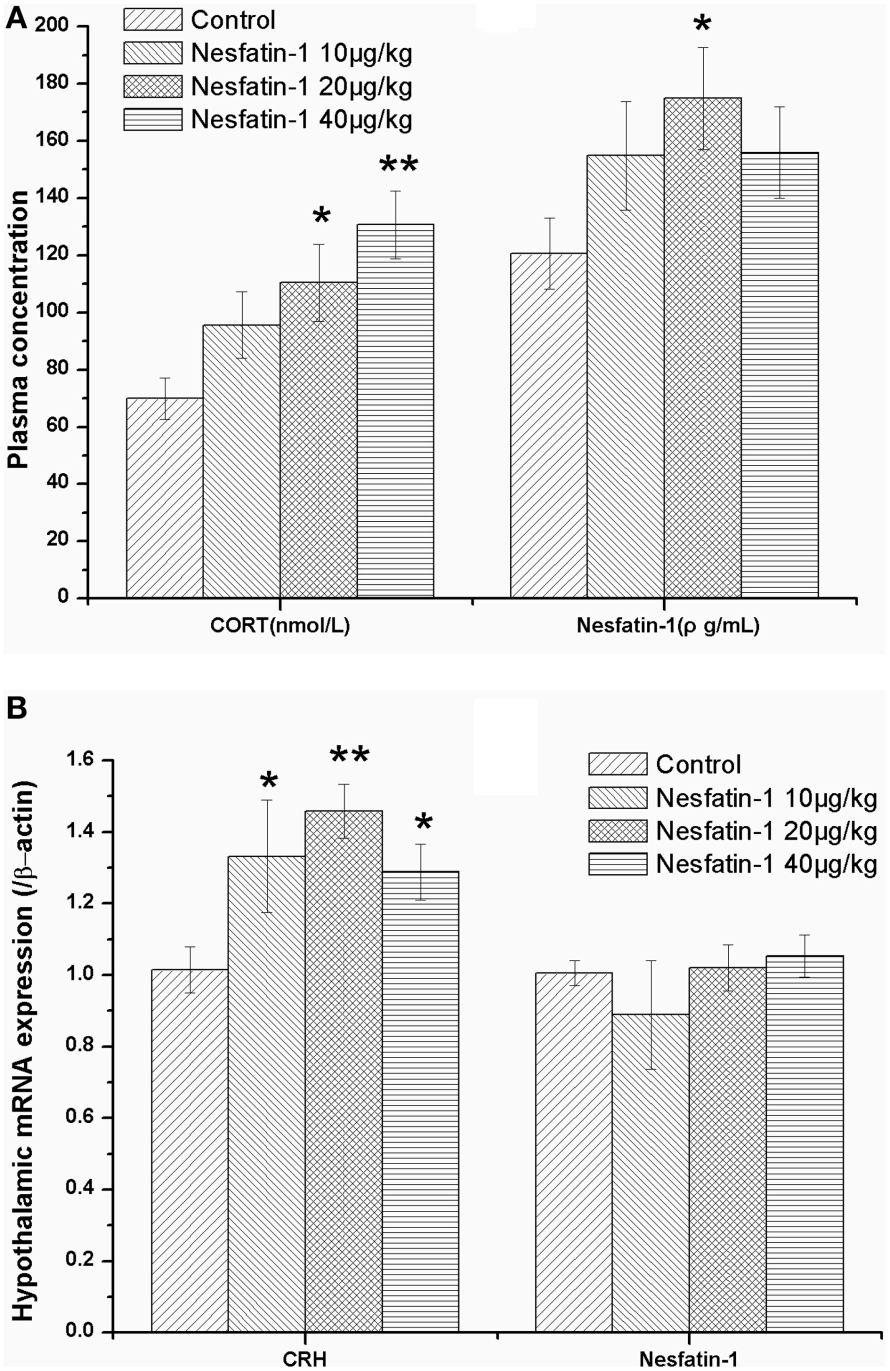
**Effect of chronic nesfatin-1 administration on the plasma concentrations of CORT and nesfatin-1 and the hypothalamic mRNA expression of CRH and nesfatin-1 in rats**. The data are presented as the mean ± SEM. Nesfatin-1 treated rats exhibited hyperactivity of the HPA axis, as indicated by the elevated serum CORT concentration **(A)** (*n* = 10 for each group) and the CRH mRNA expression in the hypothalamus **(B)** (*n* = 5 for each group). Although the plasma nesfatin-1 concentration increased in the nesfatin-1 (20 μg/kg) group **(A)**, the hypothalamic mRNA expression of nesfatin-1 did not change after 3-weeks of consecutive administration of nesfatin-1 **(B)**. ^*^P < 0.05 and ^**^P < 0.001 compared with the control group.

As expected, the plasma CORT concentration (Figure 4A) and hypothalamic expression of CRH mRNA (Figure 4B) were both significantly increased after the 3-weeks period of nesfatin-1 administration (10, 20, 40 μg/kg) (CORT: *F* = 4.741, *P* = 0.007; CRH: *F* = 4.574, *P* = 0.014) Although the mRNA expression of NUCB2/nesfatin-1 in the hypothalami (Figure 4B) was not different among the four groups (*F* = 0.743, *P* = 0.539), the chronic administration of nesfatin-1 (20 μg/kg) markedly increased the plasma concentration of nesfatin-1 (Figure 4A).

The results of the Spearman correlation analysis showed that there was a dose-effect relationship between nesfatin-1 exposure and the increase in the plasma CORT concentration (*r* = 0.559, *P* < 0.001) and the hypothalamic expression of CRH mRNA (*r* = 0.436, *P* = 0.033).

### Increased mRNA expression of synaptotagmin I and synapsin I in the hypothalamus induced by chronic administration of nesfatin-1

**Figure 5:**
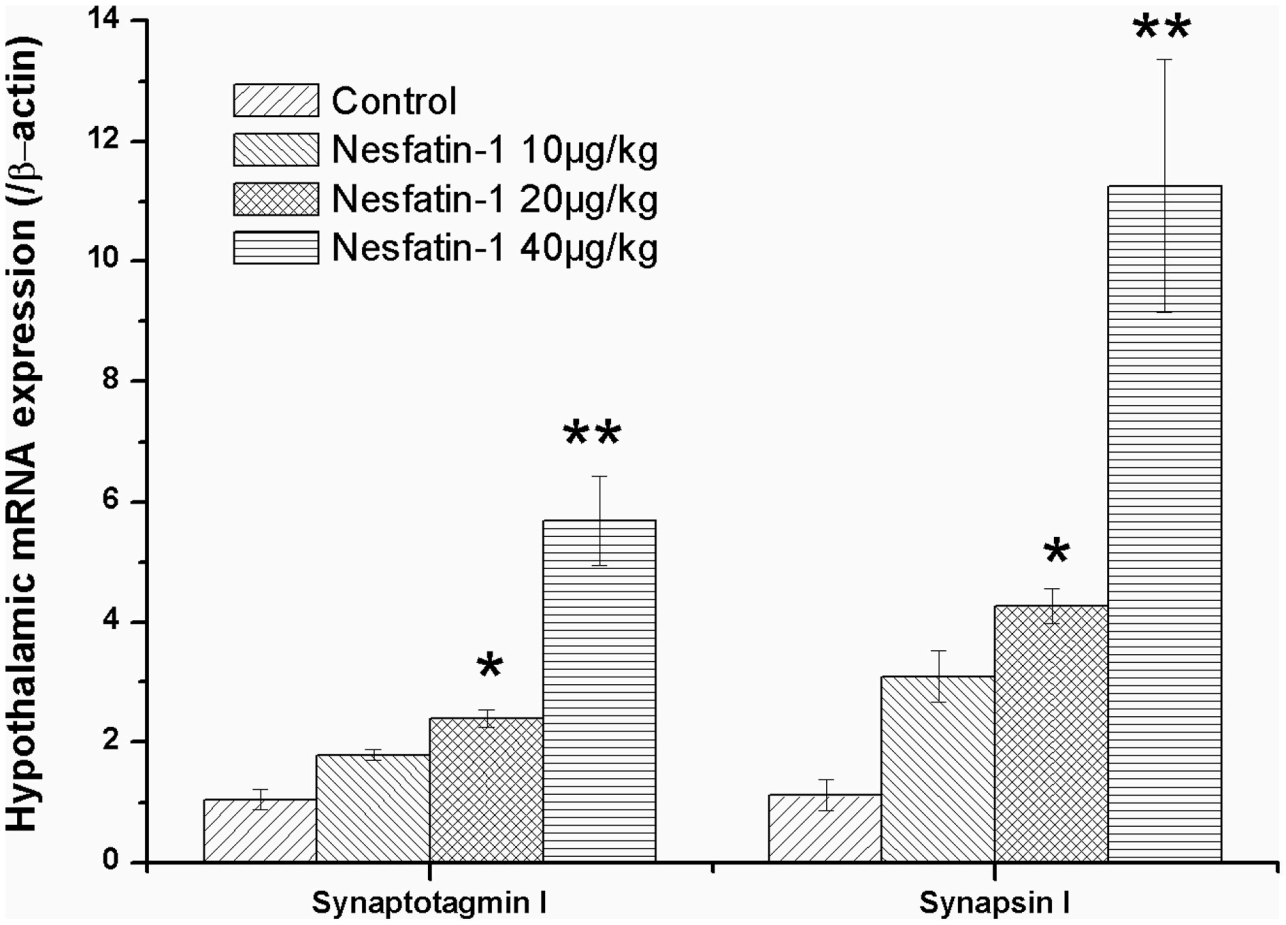
**Effect of chronic nesfatin-1 administration on the hypothalamic mRNA expression of synaptotagmin I and synapsin I in rats**. The data are presented as the mean ± SEM, with *n* = 5 for each group. The hypothalamic mRNA expression levels of synaptotagmin I and synapsin I in nesfatin-1 treated rats were up-regulated compared with those in the controls. ^*^P < 0.05 and ^**^P < 0.001 compared with the control group.

Figure 5 shows the mRNA expression of synaptotagmin I and synapsin I in the hypothalami of the rats in the different groups. After the 3-weeks of nesfatin-1 administration (20, 40 μg/kg), the hypothalamic mRNA expression levels of synaptotagmin I and synapsin I were up-regulated compared with the control group (synaptotagmin I: *F* = 9.590, *P* = 0.002; synapsin I: *F* = 6.742, *P* = 0.006).

The results of the Spearman correlation analysis showed the positive correlation between nesfatin-1 exposure and the hypothalamic expression of synaptotagmin I mRNA (*r* = 0.910, *P* < 0.001) and synapsin I mRNA (*r* = 0.877, *P* < 0.001).

### Increased plasma concentrations of IL-6 and CRP induced by the chronic administration of nesfatin-1

**Figure 6:**
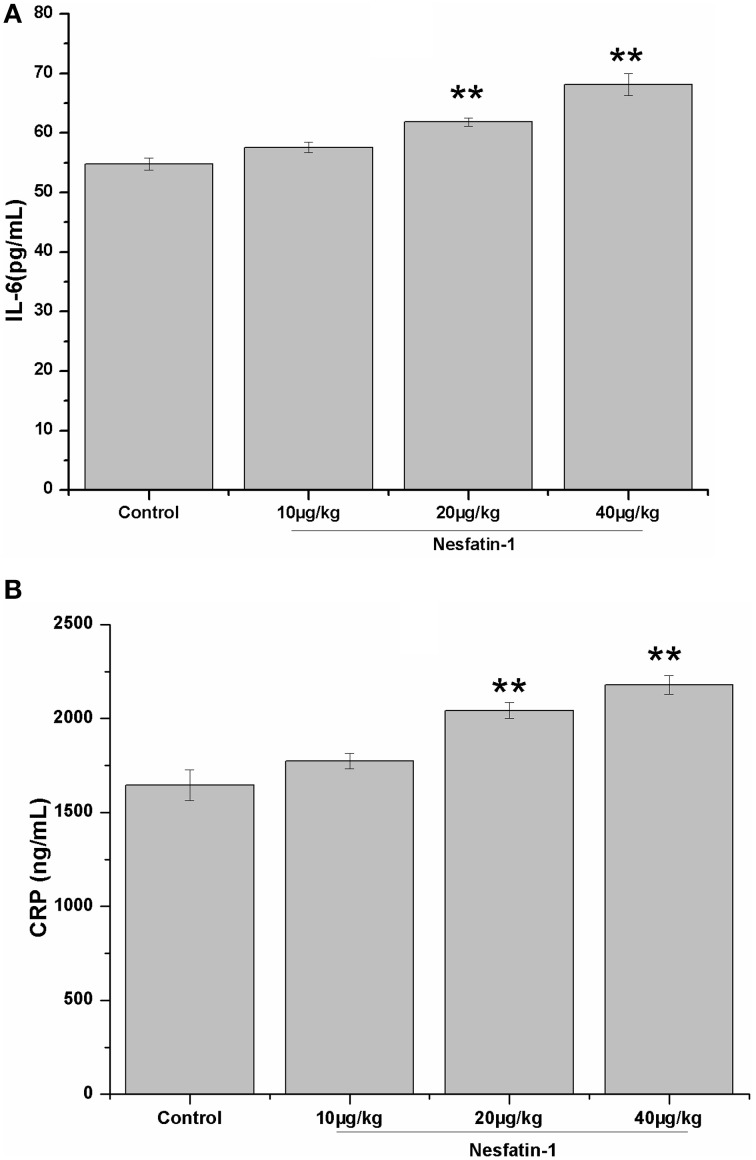
**Effect of chronic nesfatin-1 administration on the plasma concentrations of IL-6 and CRP in rats**. The data are presented as the mean ± SEM, with *n* = 10 for each group. The plasma concentrations of IL-6 and CRP in the nesfatin-1-treated rats were higher than those in the control rats. ^**^P < 0.001 compared with the control group.

Figure 6 shows the plasma concentrations of IL-6 and CRP in all groups. The 3-weeks of nesfatin-1 administration remarkably increased the plasma IL-6 (*F* = 21.775, *P* < 0.001) and CRP (*F* = 20.569, *P* < 0.001) levels in a dose-dependent manner (IL-6: *r* = 0.801, *P* < 0.001; CRP: *r* = 0.834, *P* < 0.001). The results of the Pearson correlation analysis showed that the plasma IL-6 and CRP concentrations were both positively correlated with the immobility (IL-6: *r* = 0.329, *P* = 0.041; CRP*: r* = 0.362, *P* = 0.023) in the FST and the plasma CORT level (IL-6: *r* = 0.390, *P* = 0.016; CRP*: r* = 0.554, *P* < 0.001). The plasma concentration of CRP, but not IL-6, was negatively correlated with the frequencies of rearing (CRP*: r* = −0.359, *P* = 0.025; IL-6: *r* = −0.267, *P* = 0.101) and grooming (CRP*: r* = −0.405, *P* = 0.011; IL-6: *r* = −0.285, *P* = 0.079) in the last OFT.

Although, the sample size is limited, positive relationships were also found between the plasma CRP and IL-6 concentrations and the hypothalamic mRNA expression of synaptotagmin I (CRP*: r* = 0.646, *P* = 0.008; IL-6: *r* = 0.641, *P* = 0.007) and synapsin I (CRP*: r* = 0.635, *P* = 0.008; IL-6: *r* = 0.629, *P* = 0.009).

## Discussion

In the present study, we first demonstrated that an intraperitoneal administration of a single dose of nesfatin-1 increased the immobility of rats in the FST and induced the hyperactivity of the HPA axis. Based on these results, together with the fact that chronic administration is a route delivery method in clinic, we explored the effect of chronic nesfatin-1 treatment on the depression-like behavior of rats and the underlying mechanism. The results showed that after chronic nesfatin-1 administration, the rats exhibited increased immobility in the FST, and decreased activity and exploratory behavior in the OFT, which were accompanied by a hyperactive HPA axis, as indicated by the increased levels of plasma CORT and hypothalamic CRH mRNA. Increased mRNA expression levels of synaptotagmin I and synapsin I in the hypothalami were also found in the nesfatin-1-treated rats. Moreover, the chronic administration of nesfatin-1 elevated the plasma concentrations of CRP and IL-6, which were correlated with the lower activity and despair behavior, the HPA hyperactivity, and the changes in the expression of the synaptic proteins.

The stress-associated activation of NUCB2/nesfatin-1 neurons, together with nesfatin-1's central functions in the brain indicated its potentially significant role in the stress adaptation response. Results from human studies have provided more data linking nesfatin-1 to the onset of depression (Ari et al., 2011; Bloem et al., 2012). In the present study, our results showed that the intraperitoneal administration of a single dose of nesfatin-1 and the chronic administration of nesfatin-1 increased the immobility in the FST and that the chronic administration of nesfatin-1 decreased the activity and exploratory behavior of the rats in the OFT. Moreover, when given chronically, nesfatin-1 was dose-dependently correlated with the despair-like behavior. In stress-induced depression studies, lowered activity and behavioral despair, as indicated by the reduced moving distance and low levels of rearing and grooming in the OFT, and increased immobility in the FST are often used as indices of a depressive-like state (Izumi et al., 1997; Ge et al., 2013). Thus, the present study emphasizes the important role of nesfatin-1 in the stress-related response. We are not exactly sure about the reason why the performance of the nesfatin-1-treated rats in FST were similar after acute and chronic administration, although it may be interpreted partly by the possibility that steady state plasma concentration of nesfatin-1 reach when chronically treated. However, we could not provide effective pharmacokinetic parameters of nesfatin-1 to back up this hypothesis in the present study.

Surprisingly, in the sucrose preference test, the sucrose preference index was not significantly different between the nesfatin-1-treated rats and the controls. However, our results indicated that the rats in the nesfatin-1-treated group drank less plain water, which was partly consistent with the results reported by Yosten et al. that ICV nesfatin-1 significantly inhibited the drinking response of rats, which was independent of its anorexigenic effect (Yosten et al., 2012).

Hyperactivity of the HPA axis is thought to play an important role in the etiology of depression (Swaab et al., 2005; Ge et al., 2014). In the present study, our results showed that the intraperitoneal administration of nesfatin-1 in a single dose or chronically could induce the hyperactivity of the HPA axis, as indicated by the increases in the plasma CORT concentration and the hypothalamic expression of CRH mRNA, which was consistent with the effect of centrally administered nesfatin-1 (Konczol et al., 2010; Yoshida et al., 2010). Moreover, when nesfatin-1 was administered chronically, the results of the Spearman correlation analysis demonstrated a dose-effect relationship between nesfatin-1 exposure and the hyperactivity of the HPA axis. These findings indicated that the effects of nesfatin-1 were associated with the HPA axis.

Synaptic vesicle proteins have been identified as possible factors involved in the pathophysiology of psychiatric disorders including depression. Region specific changes in the expression of synaptotagmin I and synapsin I have been reported to be induced by both stress and antidepressant (Dagyte et al., 2011; Müller et al., 2011), suggesting a regulatory role of synaptotagmin I and synapsin I in the pathogenesis of depression. In the present study, our results showed that the mRNA expression levels of synaptotagmin I and synapsin I were increased in the hypothalamus following nesfatin-1 treatment. Given the findings that Ca^2+^ binding synaptotagmin I functions as a calcium sensor in the regulation of neurotransmitter release and that the primary role of synapsin I is the regulation of vesicle turnover, it is predicted that changes in their expression will affect synaptic plasticity. Our data thus, indicated that modulation of synaptic function may be involved in the depression-like behavior induced by nesfatin-1. Although the mechanism remains unknown, it may be partly ascribed to the hyperactivity of the HPA axis induced by nesfatin-1, based on the findings that synapsin-Ia/Ib and synaptotagmin are the effectors of the glucocorticoid-signaling cascade (Revest et al., 2010; Wuwongse et al., 2013).

The multicausal etiology of depression may also be explained by activated immune-inflammatory pathways (Maes et al., 2014). IL-6, together with CRP, has received much attention in the pathogenesis of depression (Liu et al., 2014; Wium-Andersen et al., 2014). An increased IL-6 concentration has been detected in depressed patients (Maes et al., 2001) and animals (Voorhees et al., 2013), and the administration of IL-6 could produce depressive-like behaviors in mice (Sukoff Rizzo et al., 2012). Moreover, it has been reported that increased IL-6 levels contributed to heightened HPA axis activity (Leonard and Maes, 2012; Maes et al., 2014), and significant positive correlation was found between the cortisol and CRP levels (Cubala and Landowski, 2014). In the present study, the chronic administration of nesfatin-1 increased the plasma concentrations of IL-6 and CRP, which were positively correlated with the despair behavior and the plasma CORT concentration. These results indicated that IL-6 and CRP may play an important role in the pathogenesis of nesfatin-1 induced depression-like changes. Together with the results of the Pearson correlation test, it is rational to hypothesize that the immune-inflammatory activation is the central hub that linked the imbalance of synaptic vesicle proteins and the depression-like behavior induced by nesfatin-1administration.

Although the original function identified for nesfatin-1 was its inhibition of dark cycle food intake (Mortazavi et al., 2015), it has been reported that the peripheral infusion of nesfatin-1 has no effect on dark or light cycle food intake or body weight (Li et al., 2013). Consistent with this finding, there was no significant difference in bodyweight between the nesfatin-1-treated rats and the controls in the present study.

Conflicting results have also been reported regarding the effects of nesfatin-1 on glucose metabolism and insulin secretion. It has been demonstrated that the peripheral infusion of nesfatin-1 could significantly affect glucose metabolism through a direct peripheral mechanism to increase insulin secretion and insulin sensitivity (Gonzalez et al., 2011; Li et al., 2013) but both increased and decreased plasma levels of nesfatin-1 were reported in patients with newly diagnosed type 2 diabetes mellitus (Li et al., 2010; Zhang et al., 2012). However, in the present study, a consecutive 3-week intraperitoneal treatment with nesfatin-1 did not affect the plasma concentrations of glucose and insulin in rats (Supplementary Materials). This discrepancy may be the result of differences in the study designs and experimental conditions. In the case of lipid metabolism, although the plasma concentrations of VLDL and TC increased after the chronic administration of nesfatin-1 (40 μg/kg), other lipid-associated indicators remained unchanged in the present study (Supplementary Materials).

There are several limitations of this study that should be noted. First, the design of the behavior tests was imperfect. For example, the locomotor activity was only measured via the OFT, and we did not test whether the behavior changes could be reversed by treatment with an antidepressant. Therefore, detailed investigations should be conducted in the future. Second, the plasma and brain nesfatin-1 levels in the acute experiment were not measured, which may help determine whether nesfatin-1 crossed the brain-blood barrier to better understand the mechanisms underlying the effects of nesfatin-1 on behavior. Third, the parameters evaluated in the acute treatment experiment were too limited to compare the difference of the effect between a single dose and chronic administration. Indeed, the acute nesfatin-1 treatment was only a preliminary experiment to investigate the possible link between nesfatin-1 and depression. Based on the findings that nesfatin-1 given in a single dose could increase the immobility in the force swimming test, the CORT concentration, and the CRH expression, we conducted the scheme of chronic administration of nesfatin-1.

In conclusion, our results demonstrated that the intraperitoneal administration of nesfatin-1 in a single dose or chronically resulted in despair behavior and HPA axis hyperactivity. When given chronically, the depression-like effect of nesfatin-1 is correlated with an increase in plasma concentrations of IL-6 and CRP, which is accompanied by an imbalance in the mRNA expression of synaptotagmin I and synapsin I in the hypothalamus.

## Author contributions

Associate professor JG and professor FC designed the study, and wrote the protocol and the first draft of the manuscript. Prof. JG and Dr. YX managed the literature searches and the statistical analyses. YX, GQ, YP, CZ, XL, LL, and ZW performed animal model experiments. All authors contributed to and have approved the final manuscript.

## Funding

Funding for this study was provided by the Natural Science Foundation of China (81401122), Specialized Research Fund for the Doctoral Program of Higher Education of China (20133420120005), Training Programme Foundation for the Talents by Anhui Education Commission (KJ2014RC004), and the Natural Science Foundation of Anhui Province of China (1408085MH154).

### Conflict of interest statement

The authors declare that the research was conducted in the absence of any commercial or financial relationships that could be construed as a potential conflict of interest.
