# Supplementary material for: Depression-like Behavior Induced by Nesfatin-1 in Rats: Involvement of Increased Immune Activation and Imbalance of Synaptic Vesicle Proteins
Source: Front Neurosci. 2015 Nov 10;9:429. doi: 10.3389/fnins.2015.00429 (PMC4639614; doi:10.3389/fnins.2015.00429)

Supplementary materials:

Plasma concentrations of Blood glucose (BG), insulin (INS), total cholesterol (TC), low density lipoprotein (LDL), triglycerides (TG), free fatty acid (FFA), high density lipoprotein (LDL), very low density lipoprotein (VLDL), and C-reactive protein (CRP) were detected using commercially available ELISA kits (Yuanye Biotech. Co., LTD, Shanghai, China). As shown in Fig. S1, the plasma concentrations of BG, INS, LDL, VLDL, TG, FFA, and HDL were not significantly changed after chronic administration of nesfatin-1, although the plasma TC level was higher in nesfatin-1 (40 μg/kg) group than that in the control group.

**Figure Legends**

**Fig. S1** Effect of chronic nesfatin-1 administration on the plasma glucose and lipid parameters in rats

The data are presented as the mean ± SEM, with n = 10 for each group. the plasma concentrations of BG, INS, LDL, VLDL, TG, FFA, and HDL were not significantly changed after chronic administration of nesfatin-1, although the plasma TC level was higher in nesfatin-1 (40 μg/kg) group than that in the control group.

**P* < 0.05 and ***P* < 0.001 compared with the control group.

**Fig. S1**

**
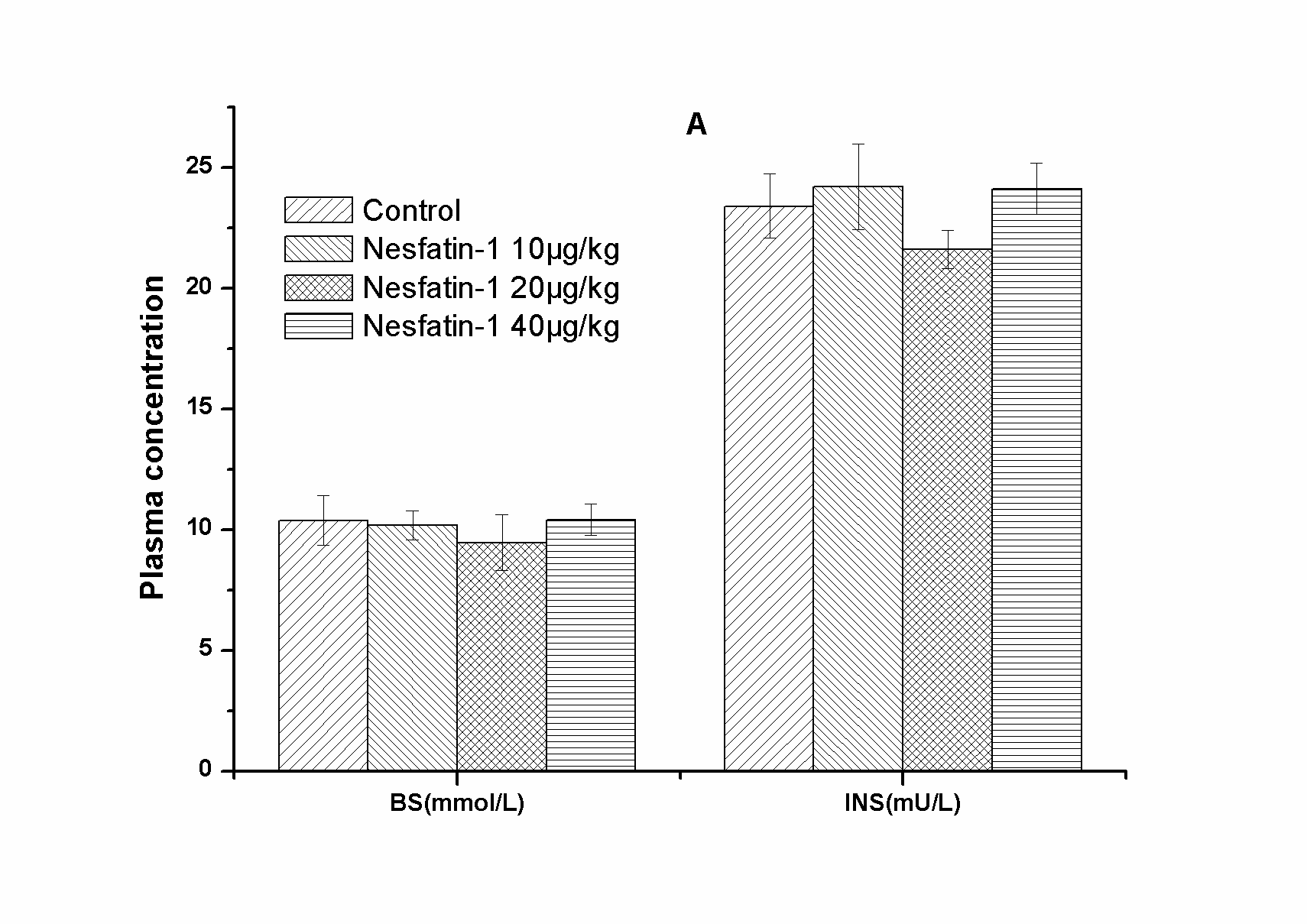
**


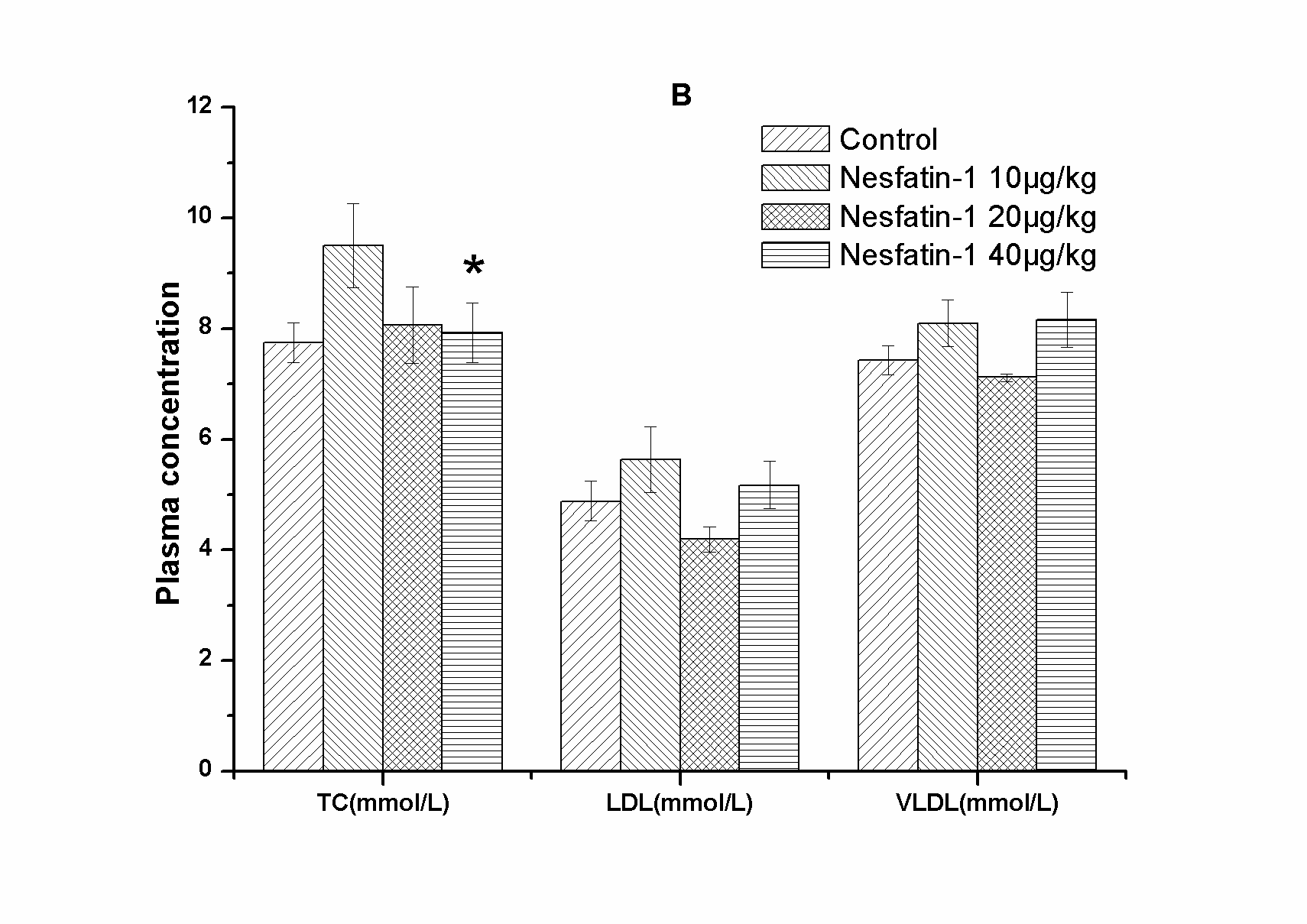


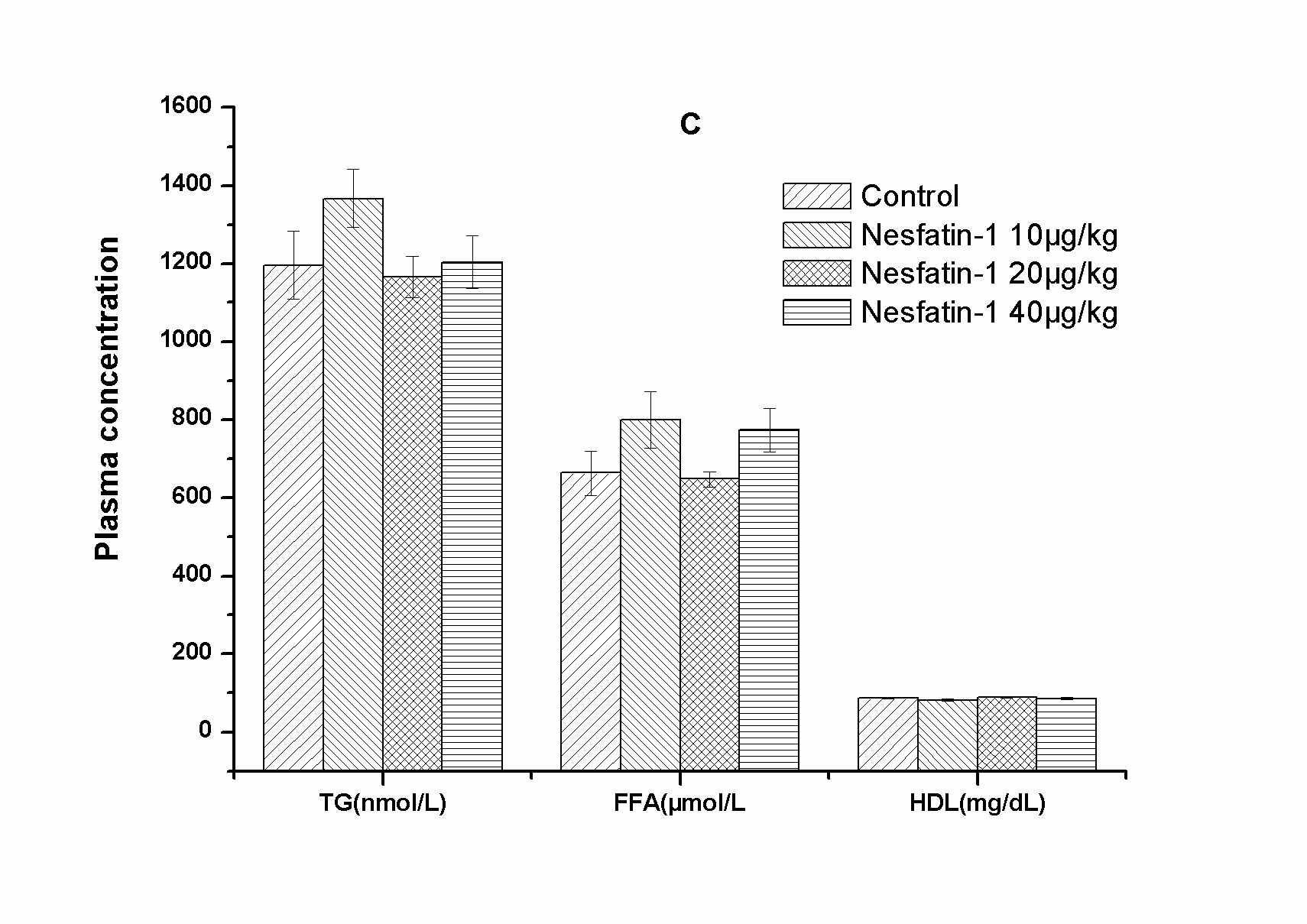

Supplement: Supplementary file 1 [file DataSheet1.DOC]
